# Heterogeneous network flow and Petri nets characterize multilayer complex networks

**DOI:** 10.1038/s41598-022-07249-6

**Published:** 2022-03-03

**Authors:** Alma Ademovic Tahirovic, David Angeli, Goran Strbac

**Affiliations:** 1 Department of Electrical and Electronic Engineering, Imperial College London, London, SW7 2AZ UK; 2 Department of Information Engineering, University of Florence, 50139 Florence, Italy

**Keywords:** Information theory and computation, Computational science, Computer science, Information technology

## Abstract

Interacting subsystems are commonly described by networks, where multimodal behaviour found in most natural or engineered systems found recent extension in form of *multilayer networks*. Since multimodal interaction is often not dictated by network topology alone and may manifest in form of cross-layer information exchange, *multilayer network flow* becomes of relevant further interest. Rationale can be found in most interacting subsystems, where a form of multimodal flow across layers can be observed in e.g., chemical processes, energy networks, logistics, finance, or any other form of *conversion process relying on the laws of conservation*. To this end, the formal notion of *heterogeneous network flow* is proposed, as a multilayer flow function aligned with the theory of network flow. Furthermore, dynamic equivalence is established with the framework of *Petri nets*, as the baseline model of concurrent event systems. Application of the resulting multilayer Laplacian flow and flow centrality is presented, along with graph learning based inference of multilayer relationships over multimodal data. On synthetic data the proposed framework demonstrates benefits of multimodal flow derivation in critical component identification. It also displays applicability in relationship inference (learning based function approximation) on multimodal time series. On real-world data the proposed framework provides, among others, multimodal flow interpretation of U.S. economic activity, uncovering underlying empirical steady state probability distribution, as well as inherent network (economic) robustness.

## Introduction

Networks represent a common tool used to describe interacting subsystems, by formalizing their physical or abstract connections. Modes of connectivity are often regarded as mono-semantic, however, true interaction in most natural or engineered systems is frequently multimodal in nature. The inability to describe such systems by traditional networks motivated extension in form of *multilayer networks*^1,2^, based on applications found in sociology and psychology^3–8^, chemistry^9,10^, and physics^11–15^. This attempt to develop a framework and generalize tools from network science to study multilayer complex systems is only recent^2^. Some common formalisms falling under the framework of multilayer networks involve multiplex networks (single type of nodes, multiple types of edges)^16,17^, networks of networks (multiple types of networks, connected by partially dependent node or network pairs)^18^, or heterogeneous networks (multiple types of networks, connected by distinct types of nodes and edges)^19–22^. A comprehensive survey of common concepts falling under the framework of multilayer networks is provided in works such as^2,23^. Some of these networks e.g., heterogeneous networks, are furthermore formally classified as layer-disjoint networks, a term meant to emphasize that each node of the network associates to a single layer (single type) only^2^.

Beyond its topological aspect, interaction in interconnected subsystems is often characterized by some form of evolution process, which under steady state conditions can be described as a *network flow*. Network flow in multilayer settings has so far been studied in multiplex networks^24^, coupled cell networks (multiple types of nodes and edges, but requiring single-type edges for bidirectional flow)^25^, or has otherwise been regarded in the context of a random walk or diffusion movement in a single-mode sense^26,27^. A formal *theory of network flow*^28,29^, satisfying conditions of both conservation and coupling of flow across different network semantics, has so far not been proposed in the context of multilayer networks, or within the framework presented in this paper. Besides the merit of a unified formal treatment, the rationale lies in an underlying physical interpretability found in most interacting subsystems, where a form of multimodal flow across layers can be observed in e.g., chemical processes, energy networks, logistics, finance, or any other form of *conversion process relying on the laws of conservation*. Some real-world examples of interacting subsystems with multilayer network structure involve, multi-carrier energy networks, financial networks, and transportation networks, to name a few. To this end, the formal notion of *heterogeneous network flow* is proposed, as a multilayer flow function aligned with the theory of network flow^28,29^. A dynamic equivalence with the framework of *Petri nets*^30,31^ is established, as the baseline model of concurrent event systems, relating to continuous timed processes^32–34^ and associated network flow^35^. The construction enables flattening of the multilayer relationship structure, while retaining physical interpretability, as the proposed correspondence is reversible. The Petri net flow relations are here extended, to possibly incorporate both fundamental equations of balance^36^, namely: *flow balance*, which is integral to the Petri net model, and *node potential balance* (cycle space condition), which may arise in relation to specific application domains. Overall, where a multilayer network represents a generalization over the classic definition of a graph or network, the proposed framework represents a generalization over the notion of a network flow and node relationship (whenever, due to semantics, both connectivity and conversion of data are crucial). As such, the proposed framework enables derivation of a *layered relationship structure* (corresponding to connectivity and conversion of node data), as opposed to a classic *flat relationship structure* (corresponding to connectivity of node data only). Applications of the resulting multilayer Laplacian flow and flow centrality are presented, along with graph learning based inference of multilayer relationships over multimodal data.

The remainder of this paper is organized as follows. In “Background concepts” an overview of mathematical preliminaries is presented. The proposed methodology is introduced under “Proposed framework”, while illustration of possible applications is presented in “Illustrative examples”. In “Discussion and outlook﻿” key findings and future work are summarized, while concluding remarks are presented in “Conclusions”.

## Background concepts

To aid further understanding of the proposed framework, a brief introduction of background concepts is presented, including basic terminology and notation. The formal notions of graphs, network flow, multilayer networks and Petri nets are introduced individually, by recalling some relevant definitions.

### Graphs

A *graph* *G* is considered an ordered tuple $G=(V,E)$, with a vertex set *V* of cardinality *n* (graph order) and edge set *E* of cardinality *m* (graph size). The edge set *E* is a subset $E \subseteq [V]^{2}$, where $[V]^{2}$ denotes all two-element subsets of *V* defining distinct pairs of adjacent vertices or nodes, incident to their relevant edge. An adjacency matrix $A_{m} \in {\mathbb {R}}^{(n \times n)}$ is a matrix encoding among all distinct vertex pairs of a graph, those which belong to an edge set. An incidence matrix $A_{t} \in {\mathbb {R}}^{(n \times m)}$ is a matrix encoding all incident vertices and edges. The graph $G=(V,E',\mu ')$ comprising of not necessarily distinct vertex pairs is considered a *multigraph*^37^. The multigraph is defined with respect to a finite edge set $E'$, where association to pairs of nodes is taking place with respect to a map $\mu ':E' \rightarrow [V]^{2}$. The map $\mu '$ assigns to each edge two end vertices, allowing multiple edges between adjacent nodes (note that self-loops are left out in this definition). To simplify notation, a graph is always denoted as $G=(V,E)$, where a multigraph is always understood in the latter context. The graph can be weighted $\omega :E \rightarrow {\mathbb {R}}^{+}$ (${\mathbb {R}}^{+} = \{ x \in {\mathbb {R}} \mid x>0 \}$) or unweighted, where $\omega$ denotes the weighting coefficient. The graph is assumed *connected*, or otherwise decomposed into the union of its connected graph components.

A *network* *N* is a digraph or directed graph defined on an ordered tuple $N=(V,A_{N})$. A *flow network* $N_{f}$ is a digraph with additional structural properties i.e., $N_{f}=(N,c,s,t) \equiv (V,A_{N},c,s,t)$. The digraph consist of an arc set $A_{N} \subseteq V \times V$ of ordered vertex pairs or node pairs, forming directed edges or arcs $(u,v) \in A_{N}$. Node *u* denotes the initial vertex or arc tail and node *v* the end vertex or arc head. A flow network comprises furthermore of an *arc capacity function* $c:A_{N} \rightarrow {\mathbb {R}}^{+}$. It is also defined with respect to two distinguished subsets of *V*, denoted as *S* and ${\bar{S}}$, where $S \subseteq V$ defines the set of sources of $N_{f}$, and ${\bar{S}} \subseteq V$ the set of sinks. From a physical point of view, these subsets define the sets of points where flow enters or leaves a network, in form of *exogenous flow*. From a mathematical point of view, *S* and ${\bar{S}}$ are arbitrary subsets of *V* (normally disjoint, as any overlap is cancelled out as net flow). Vertices $v \in V \setminus (S \cup {\bar{S}})$ are called intermediate. Flow network $N_{f}$ can, without loss of generality, be reduced to a *flow circulation*, a single-source *s* and single-sink *t* representation by auxiliary vertices $s,t \in V$, referred to as the *environment*. Single-source *s* is incident with all source vertices $S = \{ u \mid (s,u) \in A_{N} \}$, and such that $(v,s) \notin A_{N}, \forall v \in V$. Single-sink *t* is incident with all sink vertices ${\bar{S}} = \{ v \mid (v,t) \in A_{N} \}$, and such that $(t,u) \notin A_{N}, \forall u \in V$. The transformation converts sources $u \in S \setminus \{ s \}$ and sinks $v \in {\bar{S}} \setminus \{ t \}$ to intermediate nodes, with intermediate arc set $A_{{\bar{N}}} \subseteq A_{N}$ obtained as the set of all arcs not incident with the environment. Arc capacity from and to the environment is assumed infinite. The *direction of flow* in a flow network corresponds to the direction of an arc (providing physical meaning), hence bidirectional flow is represented by two oppositely directed arcs.

### Network flow

*Network flow* is a function defined with respect to the topology of a flow network. Somewhat counter-intuitively it is not primarily concerned with the mechanics of flow, but rather with the algebraic relationships encoded in the flow process. A network flow can be considered a form of *algebraic tool*. The following definition is derived from^28,29^, chosen for interpretability (slightly adapted to the formal content of this paper), but can equivalently be given with respect to a flow circulation^37^.

#### **Definition 1**

(*Network flow*) Let *N* be a multigraph $N=(V,A_{N})$, with bidirectional arc set $A_{N}$, and let $N_{f}$ be a flow network $N_{f}=(N,c,s,t), c:A_{N} \rightarrow {\mathbb {R}}^{+}$. A *network flow* *f* is a function $f:A_{N}\rightarrow {\mathbb {R}}_{0}^{+}$, satisfying the following constraints:

(i) *Capacity constraint*: $$0 \leqslant f(i,j) \leqslant c(i,j), \forall (i,j) \in A_{N}$$,
(ii) *Conservation of flow*: $\sum _{ \{ j \mid (j,i) \in A_{N} \} } f(j,i) - \sum _{ \{ j \mid (i,j) \in A_{N} \} } f(i,j) = 0$, $\forall i \in V \setminus \{ s,t \}$,
(iii) *Complementarity constraint (direction of flow)*: $f(i,j) \cdot f(j,i) = 0, \{ \forall (i,j) \in A_{N} \mid (j,i) \in A_{N} \}$,

where *f* is a nonnegative flow with capacity constraint *c*, while $(j,i), (i,j) \in A_{N}$ correspond to inflow and outflow arcs of node $i \in V$, respectively.

The definition of network flow is here introduced with respect to the notion of *positive or negative direction* (similar to a vector), rather than positive or negative flow (providing physical meaning and some formal convenience). It is worth noting that in a classic network flow node participation is one-fold, and corresponds to conservation of flow. In the work presented in this paper this role is extended to coupling (in form of flow conversion). It is also worth pointing out that a network here is understood as an *abstract mathematical object*, where network structure encodes relationships describing certain systems or processes. This will be explained in more detail in the proposed framework section, under “Heterogeneous flow networks”.

### Multilayer networks

**Figure 1:**
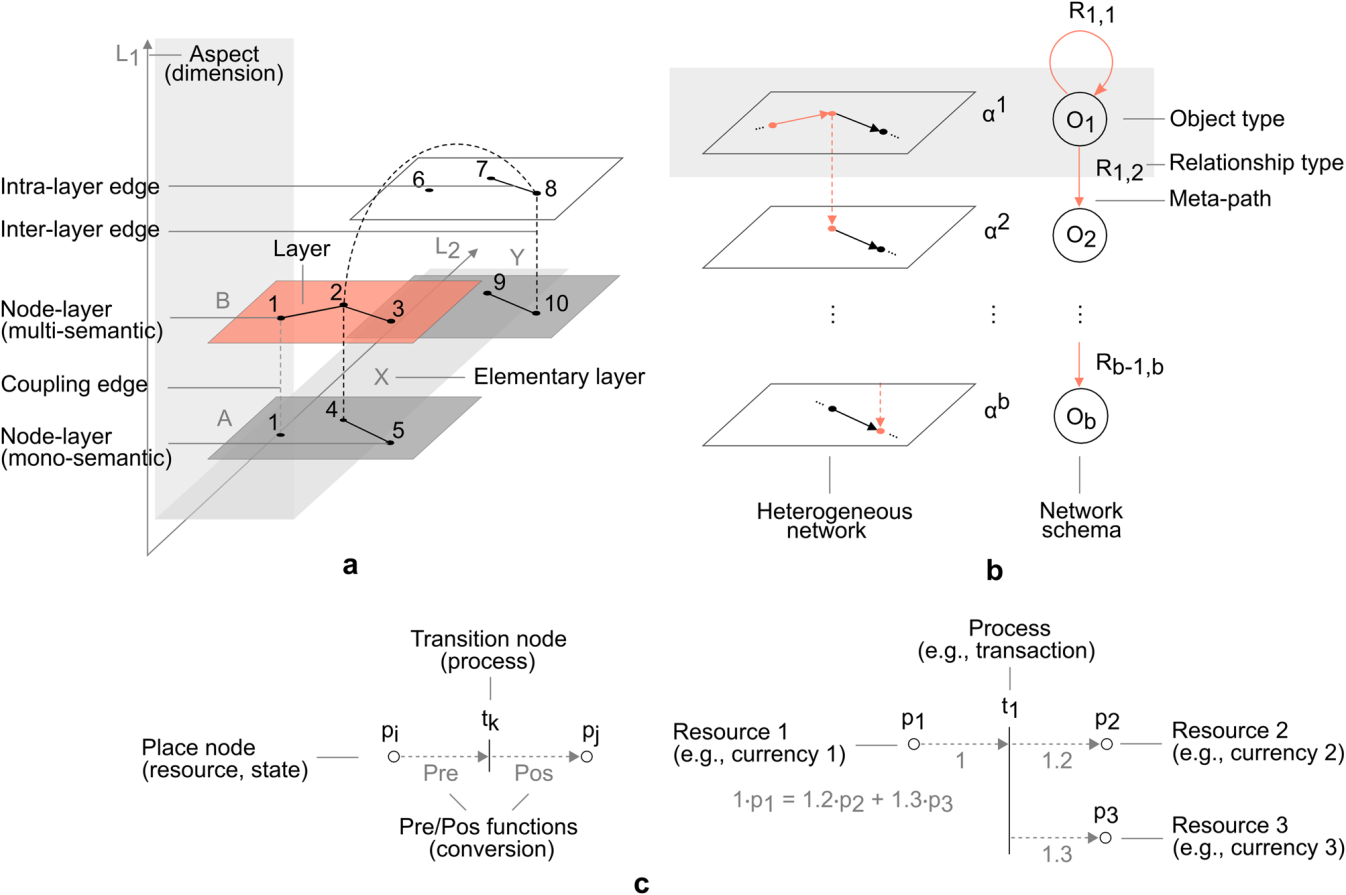
Illustration of multilayer and heterogeneous network, with Petri net. (**a**) *Multilayer network* $M = (V_{M},E_{M},V,L_{M})$, consisting of vertex set $V = \{1,\ldots ,10\}$, and layer sequence $L_{M}$ comprising of sets of elementary layers $L_{1} = \{A,B\}$, $L_{2} = \{X,Y\}$, with two aspects $d = 2$. The network consists of four layers, $\alpha ^{i} \equiv (\alpha _{1}^{i}, \alpha _{2}^{i}), i \in \{1,\ldots ,4\}: (A,X), (A,Y), (B,X), (B,Y)$, a node-layer set $V_{M} = \{(1,A,X), (4,A,X), (5,A,X), (9,A,Y), (10,A,Y), (1,B,X),\ldots , (8,B,Y)\}$, and an edge set $E_{M}$, comprising of intra-layer edge set $E_{A}$ (solid lines), inter-layer edge set $E_{C}$ (dashed lines), and coupling edge set $E_{{\bar{C}}}$ (dashed grey lines, corresponding to e.g., node-layer $(1,\alpha ^{i})$, $i \in \{1,3\}$). (**b**) *Heterogeneous (layer-disjoint) network*, with path between nodes in layers $\alpha ^{1}, \alpha ^{b}$ (left), along with network schema and *meta-path* $R_{1,1} \circ ... \circ R_{b-1,b}$, formed of relation types $R_{i,j} \in R_{T}$, between object types $O_{i}, O_{j} \in O_{T}$, $i,j \in \{ 1,\ldots ,b\}$ (right). (**c**) *Petri net*, with place nodes $p_{i}, p_{j} \in P$ (circles) interpretable as resources or system states, and transition nodes $t_{k} \in T$ (bars) interpretable as processes, along with *Pre* and *Pos* functions (arcs), interpretable as conversion ratios or weights (left) (e.g., 1 unit of resource $p_{1}$, produces 1.2 units of resource $p_{2}$, and 1.3 units of resource $p_{3}$, through process $t_{1}$ (right)).

A *multilayer network* comprises of a node set *V*, as any other network or graph. It furthermore consists of *layers* $\alpha ^{i}$, $i \in \{ 1,\ldots ,b \}$ (Fig. 1a, horizontal rectangles), where *b* denotes the total number of layers. Each layer $\alpha ^{i}$ is an array $(\alpha _{1}^{i},\ldots ,\alpha _{d}^{i})$ of *elementary layers* $\alpha _{a}^{i}$ (Fig. 1a, Cartesian axes labels, e.g., *X*, *Y*), corresponding to aspects $a \in \{ 1,\ldots ,d \}$, where *d* denotes the total number of aspects or dimensions (Fig. 1a, Cartesian axes). The layers form a *layer sequence* $L_{M} = \{ L_{a} \}_{a=1}^{d}$, consisting of sets of elementary layers $L_{a}$, where $L_{a} = \{ \alpha \mid \exists i \in \{ 1,\ldots ,b \}, \alpha _{a}^{i} = \alpha \}$ (Fig. 1a, light grey highlight). Nodes and layers form a multilayer node set $V_{M} \subseteq V \times L_{1} \times ... \times L_{d}$, comprising of *node-layers* $(u,\alpha ^{i}) \equiv (u, \alpha _{1}^{i},\ldots , \alpha _{d}^{i})$, such that *u* denotes a node $u \in V$ existing on a respective layer $\alpha ^{i}$ (Fig. 1a, e.g., node-layer $(2,\alpha ^{i})$, $i \in \{3\}$). The multilayer edge set $E_{M} \subseteq [V_{M}]^{2}$ is a two-element subsets of $V_{M}$. The *intra-layer edge set* is a set $E_{A} = \{ \{ (u,\alpha ^{i}),(v,\alpha ^{j}) \} \in E_{M} \mid \alpha ^{i} = \alpha ^{j} \}$ (Fig. 1a, solid lines), while an *inter-layer edge set* is a complementary set $E_{C} = E_{M} \setminus E_{A}$ (Fig. 1a, dashed lines). A coupling edge set is a subset $E_{{\bar{C}}} \subseteq E_{C}$, $E_{{\bar{C}}} = \{ \{ (u,\alpha ^{i}),(v,\alpha ^{j}) \} \in E_{C} \mid u = v \}$ (Fig. 1a, dashed grey lines, corresponding to e.g., node-layer $(1,\alpha ^{i})$, $i \in \{1,3\}$). One can analogously define the multilayer arc set $A_{M} \subseteq V_{M} \times V_{M}$. The tuple $G_{M} = (V_{M},E_{M})$ is referred to as the underlying *multilayer network graph*. If the graph is weighted, a weighting function $\omega _{M}: E_{M} \rightarrow {\mathbb {R}}^{+}$ is defined. The graph is otherwise referred to as unweighted. The concept of a graph or network introduced in previous subsections is what is commonly referred to as a *single-layer network* or *monoplex* in multilayer network terms. The formal definition of the presented multilayer network concept is introduced in Definition 2. A graphical illustration is presented in Fig. 1a.

#### **Definition 2**

(*Multilayer network*^1,2^) Let $L_{M}$ be a layer sequence $L_{M} = \{ L_{a} \}_{a=1}^{d}$ of sets of elementary layers $L_{a}$ for each aspect $a \in \{ 1,\ldots ,d \}$, and let $V_{M}$ be a multilayer node set $V_{M} \subseteq V \times L_{1} \times ... \times L_{d}$ of node-layers $(u,\alpha ^{i}) \equiv (u, \alpha _{1}^{i},\ldots , \alpha _{d}^{i})$, where $u \in V$ represents a node existing on a respective layer $\alpha ^{i} \equiv (\alpha _{1}^{i},\ldots , \alpha _{d}^{i})$, $i \in \{ 1,\ldots ,b \}$. Given a multilayer edge set $E_{M} \subseteq [V_{M}]^{2}$ of unordered node-layer pairs and a multilayer arc set $A_{M} \subseteq V_{M} \times V_{M}$ of ordered node-layer pairs, a *multilayer network* *M* is an ordered tuple $M = (G_{M},V,L_{M})$ defined on an underlying multilayer network graph $G_{M} = (V_{M},E_{M})$, where *M* is directed if the underlying multilayer network graph is directed i.e., $G_{M} = (V_{M},A_{M})$.

In a *heterogeneous network* all nodes associate to one specific layer or type, and can be adjacent to nodes of either the same or a different type^19^, which also applies to edges^22^. The network is formally classified as *layer-disjoint*, which emphasizes that each node of the network associates to a single layer only^2^. In the following definitions the formal notions of a layer-disjoint^2^ and heterogeneous network^19,22^ are recalled. The corresponding notions of a meta-path and information network are interpreted from^22^.

#### **Definition 3**

(*Layer-disjoint network*^2^) Let *M* be a multilayer network $M = (G_{M},V,L_{M})$, $V_{M} \subseteq V \times L_{1} \times ... \times L_{d}$, $L_{1},\ldots , L_{d} \in L_{M}$. A multilayer network *M* is said to be *layer-disjoint*, if each node $u \in V$ exists in at most one layer $\alpha ^{i}$ i.e., $(u,\alpha ^{i}), (u,\alpha ^{j}) \in V_{M} \Rightarrow \alpha ^{i} = \alpha ^{j}$.

#### **Definition 4**

(*Information network*^22^) Let *N* be a digraph $N = (V,A_{N})$, with an *object type* mapping function $\theta : V \rightarrow O_{T}$ and a *relation type* mapping function $\psi : A_{N} \rightarrow R_{T}$. An *information network* *I* is a digraph defined on an ordered tuple $I = (N,\theta ,\psi )$, such that there is a surjection $\psi (l) = (\theta (u),\theta (v))$, where each vertex $u,v \in V$ belongs to one particular object type $\theta (u),\theta (v) \in O_{T}$, and each arc $l = (u,v) \in A_{N}$ belongs to one particular relation type $\psi (l) \in R_{T}$. The digraph $T_{I} = (O_{T},R_{T})$ is a *network schema*, defined as a meta template of information network *I*.

An *information network* represents a mathematical object, establishing correspondence between a node set *V* and arc set $A_{N}$, and a set of object types $O_{T}$ and relation types $R_{T}$, respectively, such that reference to each element is preserved. The formal notion enables introduction of a heterogeneous network (Fig. 1b, left panel), where nodes and edges associate to one specific layer or type (Fig. 1b, right panel), such that there is more than one object type $O_{T}$ or relation type $R_{T}$, as introduced next (Definition 5). The notion of heterogeneity refers here to semantics (i.e., physical interpretation of disjoint layers), while other types of heterogeneity (e.g., node degree) are supported implicitly, as is any other property inherent to single-layer (classic) networks, as supported by the generalized network form, the multilayer network (where a multilayer network represents a generalization over a classic definition of a network, while a heterogeneous network represents a multilayer network class).

#### **Definition 5**

(*Heterogeneous network*^19,22^) Let *M* be a multilayer network $M = (G_{M},V,L_{M})$, $G_{M} = (V_{M},A_{M})$, and let *M* be layer-disjoint. Given an information network $H = (M,\theta ,\psi )$, with an object type mapping function $\theta : V_{M} \rightarrow O_{T}$ and a relation type mapping function $\psi : A_{M} \rightarrow R_{T}$, *H* is said to be a *heterogeneous network*, if the cardinality of the set of object types is $|O_{T}|>1$ or the cardinality of the set of relation types is $|R_{T}|>1$.

#### **Definition 6**

(*Meta-path*^22^) Let *H* be a heterogeneous network $H = (M,\theta ,\psi )$, with network schema $T_{H} = (O_{T},R_{T})$. A *meta-path* $P_{H}$ is a composite relation $R_{i,i+1} \circ ... \circ R_{j-1,j}$, $R_{i,i+1},\ldots , R_{j-1,j} \in R_{T}$, on object types $O_{i}, O_{j} \in O_{T}$, $\forall i,j \in \{ 1,\ldots ,b\}$, where $\circ$ denotes a composition operator of relations.

A network schema of a heterogeneous network corresponds to a topological projection of paths between node-layers (Fig. 1b, left panel), onto a set of composite relations between elements of object types (Fig. 1b, right panel). The formal notion of a universal path across object types (Fig. 1b, right panel) corresponds to a *meta-path*^22^. Note that an inverse relation $R_{l}^{-1}$ on a meta-path does not necessarily always exist in a general sense. A transpose relation $R_{l}^{T}$, however, does by definition.

### Petri nets

The *Petri net*^30,31^ is a model of information flow, with specific focus on concurrent event systems and topological representation of the underlying relationship structure. A Petri net is a tuple of the form $P_{N} = (P,T,Pre,Pos)$, where: *P* is a set of *n* *place* nodes, *T* is a set of *m* *transition* nodes, $Pre: P \times T \rightarrow {\mathbb {R}}_{0}^{+}$ is an incidence function specifying weights from places to transitions, and $Pos: P \times T \rightarrow {\mathbb {R}}_{0}^{+}$ is an incidence function specifying weights from transitions to places (Fig. 1c). In a physical sense, place nodes (Fig. 1c, circles) can informally be understood as resources or system states, while transition nodes (Fig. 1c, bars) can be understood as processes. The *incidence matrix* is derived as $C = Pos-Pre$. The pre and post sets are denoted as $^{\circ }u, u^{\circ }$, respectively, where $u \in P \times T$. A *marking* is a mapping of the form $Q: P \rightarrow {\mathbb {R}}_{0}^{+}$ (that can be represented by a vector, once places are ordered), which assigns to each place a nonnegative quantity called mark. A *P*-*invariant* is a vector of the form $x \in {\mathbb {R}}_{0}^{+^{n}}$, where $x^{T} C = 0$ with support set $\Vert x \Vert = \{ p_{i} \in P | x_{i}>0 \}$. A *T*-*invariant* is a vector of the form $y \in {\mathbb {R}}_{0}^{+^{m}}$, where $C y = 0$ with support set $\Vert y \Vert = \{ t_{j} \in T | y_{j}>0 \}$. The two relations encode fundamental laws of *mass conservation* (*P*-invariant), and *flow balance* (*T*-invariant)^38^.

The Petri net was initially introduced as a discrete model^30,31^, but was later on extended to a continuous Petri net (CPN)^32,33^ $\langle P_{N},Q_{0}, \lambda \rangle$, where: $Q_{0}$ is a vector of initial markings $Q_{0_{i}} = Q_{0_{i}}(p_{i})$, assigned to each place $p_{i} \in P$, $|P| = n$, and $\lambda = [\lambda _{1}, \lambda _{2},\ldots , \lambda _{m}]^{T}$ is a vector of finite transition *firing rates*, assigned to each transition $t_{j} \in T$, $|T| = m$. The mark enters a place $p_{i}$ at time $\tau$ and enables all adjacent transitions $t_{j} \in p_{i}^{\circ }$, as soon as the marking $Q_{i}$ becomes available. The marking evolution is governed by the state equation

1 $$\begin{aligned} {\dot{Q}} = C \lambda \end{aligned}$$

such that transition $t_{j} \in T$ is considered enabled, provided that one can find a rate vector $\lambda \in {\mathbb {R}}_{0}^{+^{m}}$, with $t_{j} \in \Vert \lambda \Vert$, where $[C \lambda ]_{i} \ge 0$ for all *i* such that $Q_{i} = 0$. The role of flow in a CPN is directly related to the notion of a stationary transition firing rate $\lambda$. The set of admissible firing rates at equilibrium can be computed by means of a CPN linear program^35^, which for a system of the form $\langle P_{N},Q_{0}, \lambda \rangle$, with incidence matrix *C* and a finite firing rate $\lambda$, can be formulated as

2 $$\begin{aligned} \{ \lambda \ge 0 \mid C \lambda = 0 \}. \end{aligned}$$

## Proposed framework: heterogeneous network flow (multimodal flow)

The proposed framework is introduced with respect to two novel complementary concepts, namely: the formal notion of a *heterogeneous flow network*, as the underlying network topology and multilayer equivalent of a Petri net, and *heterogeneous network flow*, as the multilayer flow function enabling derivation of multimodal flow. The framework is thereby aligned with formal requirements from complex network theory and the theory of network flow, introduced in “Background concepts”.

### Heterogeneous flow network

A *heterogeneous flow network* (HFN) is a class of layer-disjoint multilayer flow networks, endowed with structural properties aligned with the theory of network flow (Definition 1). For tractability and with slight abuse of notation, node-layers $(u,\alpha ^{i})$ are from here on denoted as $u^{i}$, given layer-disjoint nature of an HFN, which preserves the layer reference of node *u*. In the following definition the formal notion of an HFN is introduced.

#### **Definition 7**

(*Heterogeneous flow network*) Let *H* be a heterogeneous network $H = (V_{M}, A_{M}, V, L_{M}, \theta , \psi )$, where $A_{A} \subseteq A_{M}$ is a set of intra-layer arcs of weight $\omega : A_{A} \rightarrow {\mathbb {R}}^{+}$, and $A_{C} = A_{M} \setminus A_{A}$ a set of *inter-layer coupling links* of weight $k: A_{C} \rightarrow {\mathbb {R}}$. A *heterogeneous flow network* $H_{f}$ is a network of the form $H_{f} = (H,c,s,t)$, with arc capacity function $c: A_{A} \rightarrow {\mathbb {R}}^{+}$, and single-source and single-sink sets $s,t \subseteq V_{M}$, $(s \cup t) \cap V_{M}^{i} = \{s^{i},t^{i}\}$, where $V_{M}^{i} \subseteq V_{M}$ is a subset of node-layers defined with respect to layer $\alpha ^{i}$, $i \in \{1,\ldots ,b\}$, and $A_{{\bar{A}}} = \{ (u^{i},v^{i}) \in A_{A} | u^{i},v^{i} \notin s \cup t \}$ is an intermediate intra-layer arc set.

**Figure 2:**
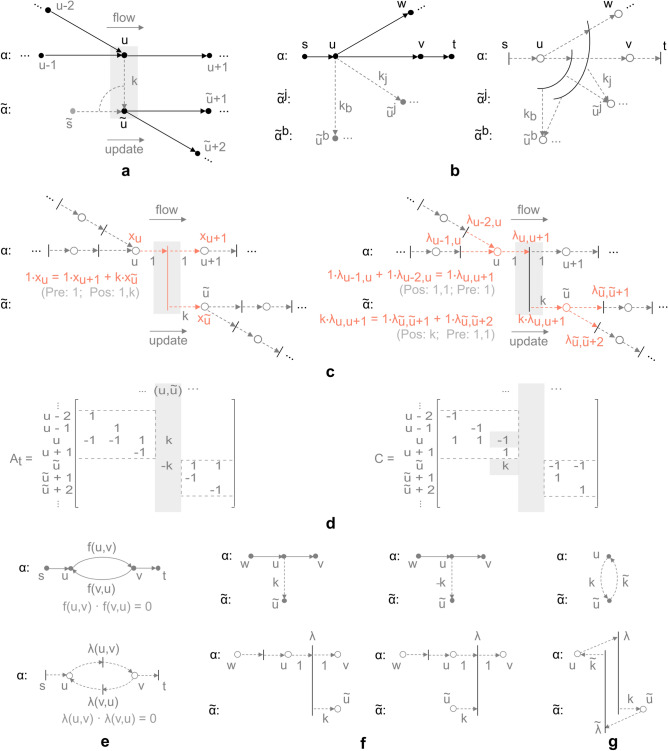
Simplified illustration of heterogeneous flow network and network transformation. (**a**) *Heterogeneous flow network* $H_{f}$ and split node *u*, split into two distinct nodes *u* and ${\tilde{u}}$, residing in two layers $\alpha$, ${\tilde{\alpha }}$, and forming link $(u,{\tilde{u}})$, which acts as exogenous arc with respect to the sink layer ${\tilde{\alpha }}$ (mapped to single-source ${\tilde{s}}$ as flow circulation). (**b**) *Transformation* of heterogeneous flow network (left-hand side), to line-graph Petri net equivalent (right-hand side), where arcs (lines) match transition nodes (bars), while nodes (dots) match places (circles) with corresponding end node transitions. Single-source and single-sink arcs, (*s*, *u*) and (*v*, *t*) , are mapped as transition nodes only, corresponding to sources and sinks of exogenous flow. (**c**) Breakdown of line-graph Petri net equivalent of network in panel a, with corresponding set of relations, where highlighted objects encode: *conservation of mass* (left-hand side), and *flow balance* (right-hand side). (**d**) Illustration of *incidence matrix* transformation, corresponding to networks in panels a and c, where $A_{t}$ corresponds to graph incidence matrix of network in panel a (unweighted, except for link $(u,{\tilde{u}})$), while *C* corresponds to Petri net incidence (coupling) matrix of network in panel c. The relationship can equivalently be written as: $[C]_{{\tilde{u}},l} = -a_{t}({\tilde{u}}, {\tilde{l}})$, if $l = (u,v) \in A_{A}$, ${\tilde{l}} = (u,{\tilde{u}}) \in A_{C}$, and $[C]_{{\tilde{u}},l} = -a_{t}({\tilde{u}},l)$, otherwise, where $(u,{\tilde{u}}) \in A_{C}$ is an inter-layer coupling of weight $k: A_{C} \rightarrow {\mathbb {R}}$, while $a_{t}(u,l) = [A_{t}]_{u,l}$ is an element of otherwise unweighted incidence matrix $A_{t}$ of $H_{f}$, and *C* the incidence matrix of $P_{f}$. (**e**) Illustration of *bidirectional arc* and corresponding Petri net transformation, where net flow flows either in positive or negative direction. (**f**) Illustration of *positive* (left-hand side) and *negative* (right-hand side) *flow conversion*, corresponding to relationship of multimodal node *u* to sink layer ${\tilde{\alpha }}$, as a form of source or sink assignment, respectively (linked to source layer firing rate $\lambda$). (**g**) Illustration of *transpose flow*, corresponding to a flow conversion with respect to complementary sink layers $\alpha , {\tilde{\alpha }}$ (linked to complementary source firing rates $\lambda , {\tilde{\lambda }}$).

The HFN is layer-disjoint in the sense that a node residing in multiple layers of an HFN has parts physically belonging to different semantic domains. The correspondence can be linked to the notion of a *split node*^29^, a network transformation converting one node *u* into *b* distinct nodes $u^{i}$, connected by objects referred to as *link*s, $(u^{i},u^{j})$, $i \ne j$, $\forall i,j \in \{1,\ldots ,b\}$ (Fig. 2a, illustrating a heterogeneous flow network $H_{f}$, with node *u* split into two distinct nodes, *u* and ${\tilde{u}}$, forming link $(u,{\tilde{u}})$). In an HFN a link corresponds to an inter-layer arc (where $A_{{\bar{C}}} = \emptyset$ i.e., the nodes reside in distinct layers and are layer-disjoint). The link constitutes an element of the composite relationship structure formed between nodes in different layers i.e., the HFN *meta-path*. The definition of an HFN meta-path is taken over from Definition 6. The link is directed due to the directed nature of a relation. In topological line-graph terms (graph dual terms), a link is defined with respect to the notion of a node. The node in an HFN meets two functional properties, namely: *conservation of flow*, in its original network flow sense, and *coupling*, in its line-graph form as a link (through flow conversion). This goes beyond the classic definition of a network flow, where a flow function does not distinguish on the role of coupling or concurrency. The HFN network flow, however, does due to the following property (Definition 8): the line-graph equivalent of a heterogeneous multilayer flow network is a Petri net (Fig. 2b).

#### **Definition 8**

(*HFN-Petri net—transformation*) Let $H_{f}$ be a heterogeneous flow network $H_{f} = (H,c,s,t)$. To any given heterogeneous network $H = (V_{M}, A_{M}, V, L_{M}, \theta , \psi )$, we associate a Petri net $P_{f} = (P,T,Pre,Pos)$ defined as follows:

(i) Place nodes:
  3 $$\begin{aligned} P = V_{M} \setminus \{s,t\} \end{aligned}$$
(ii) Transition nodes:
  4 $$\begin{aligned} T = \{t_{u,v} \mid (u,v) \in A_{A}\} \end{aligned}$$
  each corresponding to a single intra-layer arc $(u,v) \in A_{A}$;
(iii) Incidence functions:
  5 $$\begin{aligned} Pre({\tilde{u}},t_{u,v})= & {} {\left\{ \begin{array}{ll} 1, &{} \text {if } {\tilde{u}} = u\\ -k(u,{\tilde{u}}), &{} \text {if } (u,{\tilde{u}}) \in A_{C}, k(u,{\tilde{u}}) < 0\\ 0, &{} \text {otherwise} \end{array}\right. } \end{aligned}$$
  6 $$\begin{aligned} Pos({\tilde{u}},t_{u,v})= & {} {\left\{ \begin{array}{ll} 1, &{} \text {if } {\tilde{u}} = v\\ k(u,{\tilde{u}}), &{} \text {if } (u,{\tilde{u}}) \in A_{C}, k(u,{\tilde{u}}) \ge 0.\\ 0, &{} \text {otherwise} \end{array}\right. } \end{aligned}$$

Finally, we associate a function ${\bar{\lambda }}: T \rightarrow {\mathbb {R}}^{+}$, which assigns to each transition a firing rate capacity bound equivalent to *c*, and a function ${\bar{\omega }}: T \rightarrow {\mathbb {R}}^{+}$, which assigns to each transition a weight equivalent to $\omega$, such that

7 $$\begin{aligned} {\bar{\lambda }} (t_{u,v})= & {} c(u,v) \end{aligned}$$

8 $$\begin{aligned} {\bar{\omega }} (t_{u,v})= & {} \omega (u,v). \end{aligned}$$

We call Eqs. (3)–(8) an *HFN-Petri net (HFN-PN) transformation*, $T_{f}:H_{f} \rightarrow P_{f}$.

#### **Remark 1**

The transformation $T_{f}: H_{f} \rightarrow P_{f}$ defined in Definition 8, retains all information about network $H_{f}$. For Eqs. (3), (4) and (7), (8) such observation is trivial, given one-to-one correspondence between all elements of domain and range, for each relation individually. Though the single-source and single-sink sets, *s* and *t*, are not mapped to the set of all places *P*, the transformation is invertible given relation (4). The relation maps the entire intra-layer arc set $A_{A}$ to a set of all transitions *T*, making *s* and *t* the complementary node sets, relating to all transitions with no incoming or outgoing places, respectively, which is uniquely determined. For Eqs. (5) and (6) the transformation is not one-to-one. However, note that $A_{C}$ is a set of all inter-layer couplings, uniquely determined by its node set counterpart. Inter-layer links $(u,{\tilde{u}}) \in A_{C}$ correspond to reference of node *u* to each distinct layer ${\tilde{\alpha }}$ the node resides on (Fig. 2b,d), as retained by labelling of node-layers in $V_{M}$, and consequently places in *P*, which can be traced back and is uniquely determined.

The transformation in Definition 8 is invertible, within the class of Petri nets obtained by the injection (Remark 1). It is retained by the one-to-one correspondence within its image (Fig. 2b). Note that a multi-semantic split node (node residing in multiple layers) or its links, correspond to a single network element, transformed to a transition node with multiple outgoing and/or incoming arrows (as an equivalent network object). Place and transition nodes can thereby, as specified in “Background concepts”, be interpreted as e.g., resources and processes, respectively.

The transformation encodes through Eq. (1), the evolution process of a positive compartmental system $q(\phi )$^39,40^, where *q* denotes donor mass (equivalent to *Q*), and $\phi$ the flux or mass flow (equivalent to $\lambda$). Incidence matrix *C*, derived from Eqs. (5) and (6), encodes furthermore, a proportion between adjacent domains $\alpha ^{i},\alpha ^{j}$, $\phi ^{j} = k \phi ^{i}$^41,42^, where *k* denotes a coupling modulus or *conversion coefficient*. It is worth noting that a Petri net concurrently represents both, conservation and coupling relations, structurally encoded in incidence matrix *C*. A Petri net can thereby be understood as a form of mathematical object, where state space relations and physical constraints are integrated into the network structure (as in the case of a classic graph or flow network) (Fig. 2c). This property, beyond computational context (transition firing rates, marking evolution process^35^) exploited in the following subsection, provides access to a number of structural and behavioural analysis tools^38^.

### Heterogeneous network flow

A *heterogeneous network flow* is a flow function, which can be described by two types of flow processes, namely: *transfer*, a classic form of intra-layer network flow, and *coupling*, a form of inter-layer flow establishing exogenous relationship between layers in form of transformation. Though coupling may conveniently be referred to as inter-layer flow, in essence, no physical flow as in the sense of transfer is taking place. The flow represents rather an exchange or update, where relationship between inflow and outflow is not necessarily an identity (as in the case of intra-layer flow). Wherever inter-layer flow is encountered, a type of *flow conversion* is taking place, in line with respective network semantics (Definition 9).

#### **Definition 9**

(*HFN-network flow*) Let $H = (V_{M},A_{M},V,L_{M},\theta ,\psi )$ be a heterogeneous multigraph with bidirectional intra-layer arc set $A_{A} \subseteq A_{M}$. Given flow network $H_{f} = (H,c,s,t)$, a *heterogeneous network flow* $f_{h}$ is a flow function $f_{h}: A_{A} \rightarrow {\mathbb {R}}_{0}^{+}$, satisfying the following constraints:

(i) *Capacity constraint*:
  9 $$\begin{aligned} 0 \leqslant f_{h}({\tilde{u}},{\tilde{v}}) \leqslant c({\tilde{u}},{\tilde{v}}), \forall ({\tilde{u}},{\tilde{v}}) \in A_{A} \end{aligned}$$
(ii) *Conservation of flow*:
  10 $$\begin{aligned} \begin{aligned}{}&\sum \nolimits _{ \{u \mid (u,{\tilde{u}}) \in A_{C} \} } (k(u,{\tilde{u}}) \cdot \sum \nolimits _{ \{v \mid (u,v) \in A_{A} \} } f_{h}(u,v)) \\&\quad + \sum \nolimits _{ \{{\tilde{v}} \mid ({\tilde{v}},{\tilde{u}}) \in A_{A} \} } f_{h}({\tilde{v}},{\tilde{u}}) - \sum \nolimits _{ \{{\tilde{v}} \mid ({\tilde{u}},{\tilde{v}}) \in A_{A} \} } f_{h}({\tilde{u}},{\tilde{v}}) = 0, \forall {\tilde{u}} \in V_{M} \setminus \{s,t\} \end{aligned} \end{aligned}$$
(iii) *Complementarity constraint (direction of flow)*:
  11 $$\begin{aligned} f_{h}({\tilde{u}},{\tilde{v}}) \cdot f_{h}({\tilde{v}},{\tilde{u}}) = 0, \{ \forall ({\tilde{u}},{\tilde{v}}) \in A_{A} \mid ({\tilde{v}},{\tilde{u}}) \in A_{A} \} \end{aligned}$$

where $({\tilde{v}},{\tilde{u}})$, $({\tilde{u}},{\tilde{v}})$ are inflow and outflow arcs of node ${\tilde{u}}$ in layer ${\tilde{\alpha }}$, respectively, (*u*, *v*) are outflow arcs of node *u* in layer $\alpha$, and $(u,{\tilde{u}}) \in A_{C}$ are coupling links of weight *k*, participating in *flow conversion* $\sum _{ \{u \mid (u,{\tilde{u}}) \in A_{C} \} } (k(u,{\tilde{u}}) \cdot \sum _{ \{v \mid (u,v) \in A_{A} \} } f_{h}(u,v))$.

Relations (9)–(11) in Definition 9 are a generalization of relations in the definition of network flow (Definition 1). The HFN-Petri net transformation (Definition 8) enables, furthermore, a unified formal treatment of both conservation and coupling relations in a compact state space form, encoded in an abstract mathematical object. This correspondence preserves, among others, *equivalence of flow balance relations* (Theorem 1), simplifying derivation of relation (10), as presented next.

#### **Theorem 1**

(Flow balance relations—equivalence) *Let* $\lambda$ *be the vector* $[\lambda (t)]_{t \in T} = [f_{h}(u,v)]_{(u,v) \in A_{A}}$*, where* $(u,v) \in A_{A}$ *are listed according to the order chosen for transitions* $t_{u,v} \in T$ *, then for all* ${\tilde{u}} \in P$

12 $$\begin{aligned} {[}C\lambda ]_{{\tilde{u}}} = \sum \nolimits _{ \{u \mid (u,{\tilde{u}}) \in A_{C} \} } (k(u,{\tilde{u}}) \cdot \sum \nolimits _{ \{v \mid (u,v) \in A_{A} \} } f_{h}(u,v)) + \sum \nolimits _{ \{{\tilde{v}} \mid ({\tilde{v}},{\tilde{u}}) \in A_{A} \} } f_{h}({\tilde{v}},{\tilde{u}}) - \sum \nolimits _{ \{{\tilde{v}} \mid ({\tilde{u}},{\tilde{v}}) \in A_{A} \} } f_{h}({\tilde{u}},{\tilde{v}}). \end{aligned}$$

#### **Proof**

To prove statement (Eq. 12), let us begin with the left-hand side of the relation. By definition of incidence matrix *C*, the construction can be rewritten as $C \lambda = Pos \lambda - Pre \lambda$, where *Pos* is an incidence function specifying weights from transitions to places, and *Pre* an incidence function specifying weights from places to transitions. Based on Eqs. (3)–(6), the construction can be rewritten in expanded form as

$$\begin{aligned} {[}Pos \lambda ]_{{\tilde{u}}}= & {} \sum \nolimits _{ \{{\tilde{v}} \mid t_{{\tilde{v}},{\tilde{u}}} \in T \} } \lambda (t_{{\tilde{v}},{\tilde{u}}}) + \sum \nolimits _{ \{u \mid (u,{\tilde{u}}) \in A_{C} \} } (k(u,{\tilde{u}}) \cdot \sum \nolimits _{ \{v \mid t_{u,v} \in T \} } \lambda (t_{u,v})) \\ {[}Pre \lambda ]_{{\tilde{u}}}= & {} \sum \nolimits _{ \{{\tilde{v}} \mid t_{{\tilde{u}},{\tilde{v}}} \in T \} } \lambda (t_{{\tilde{u}},{\tilde{v}}}) - \sum \nolimits _{ \{u \mid (u,{\tilde{u}}) \in A_{C} \} } (k(u,{\tilde{u}}) \cdot \sum \nolimits _{ \{v \mid t_{u,v} \in T \} } \lambda (t_{u,v})) \end{aligned}$$

for $\forall {\tilde{u}} \in P$, producing for $[Pos \lambda ]_{{\tilde{u}}} - [Pre \lambda ]_{{\tilde{u}}}$ the following set of relations

$$\begin{aligned} {[}C\lambda ]_{{\tilde{u}}} = \sum \nolimits _{ \{u \mid (u,{\tilde{u}}) \in A_{C} \} } (k(u,{\tilde{u}}) \cdot \sum \nolimits _{ \{v \mid t_{u,v} \in T \} } \lambda (t_{u,v})) + \sum \nolimits _{ \{{\tilde{v}} \mid t_{{\tilde{v}},{\tilde{u}}} \in T \} } \lambda (t_{{\tilde{v}},{\tilde{u}}}) - \sum \nolimits _{ \{{\tilde{v}} \mid t_{{\tilde{u}},{\tilde{v}}} \in T \} } \lambda (t_{{\tilde{u}},{\tilde{v}}}), \forall {\tilde{u}} \in P \end{aligned}$$

which for $\lambda (t_{u,v})=f_{h}(u,v)$ returns (12). $\square$

#### **Remark 2**

For a flow network $H_{f}$ associated to Petri net $P_{f}$ by transformation $T_{f}: H_{f} \rightarrow P_{f}$ and relation (12), the following statements hold: (i) $f_{h}$ fulfils condition (9) iff $0 \le \lambda \le {\bar{\lambda }}$, following from the definition of firing rate capacity bound (Eq. 7); (ii) $f_{h}$ fulfils condition (10) iff the corresponding $\lambda$ vector fulfils $C \lambda = 0$; (iii) if there exists a firing rate $\lambda$ such that conditions (9) and (10) are satisfied, then there exists a firing rate ${\tilde{\lambda }}$ such that conditions (9)–(11) are satisfied as well, and vice versa, as fulfilled by the standard construction ${\tilde{\lambda }}(t_{u,v}) = \max \{ 0, \lambda (t_{u,v}) - \lambda (t_{v,u}) \}$; (iv) solutions of ${\dot{q}} = {\dot{q}}(f_{h})$, where ${\dot{q}}(f_{h})$ corresponds to the left-hand side of relation (10), are solutions of ${\dot{Q}} = C \lambda$ given Eq. (12), where ${\dot{q}}$ relates to instantaneous flow balance at each compartment ${\tilde{u}}$ of a positive compartmental system^39^, with exogenous flow determined by all terms relating to arc sets $A_{C}$ and $A_{A} \setminus A_{{\bar{A}}}$, while remaining flow balance is determined by all residual terms. The relationship proves HFN-PN *dynamic equivalence*.

From Theorem 1 and Remark 2, it can be deduced that stationary firing rate $\lambda$ of a Petri net directly corresponds to multilayer network flow $f_{h}$ of an HFN. Incidence matrix *C* is bidirectional, with direction encoded in oppositely facing transitions (Fig. 2e). The link coupling direction may thereby exhibit two distinct forms, where *positive* or *negative flow conversion* (Fig. 2f) encodes the relationship of a multimodal node with each layer (as a form of source or sink assignment, respectively). *Transpose flow* (Fig. 2g), on the other hand, refers to a complementary relationship between participating layers, in the sense of the notion of a transpose relation. Incidence matrix *C* ultimately encodes relationships between nodes in different layers, with network flow acting as a form of algebraic tool, where time complexity of relation (12) rests on matrix-vector multiplication and is at most *O*(*nm*) , such that *n* is the graph order and *m* the graph size.

Overall, the key property of the proposed framework lies in the ability to derive multimodal flows across different types of network layers, in a compact unified form. Inter-layer flow conversion takes place across network links, which act as exogenous control flows with respect to the sink layer. The multimodal flows are implicitly either coupled (across layers) or uncoupled (in intra-layer flow derivation, specifically with regard to cycle space conditions, as presented next). This property lends some new perspective to network interdependency assessment, such as e.g., with respect to flow based centrality^43–45^, system stability or reachability of states^38^. What is important to note is that the proposed framework satisfies concurrently conditions of conservation and coupling of flow across network semantics. This is in contrast to current state-of-the-art, as presented in e.g.^26,27,46^, where the main processes studied to date relate to epidemic spreading and single-mode information diffusion^8,47,48^. This renders the proposed framework more applicable to certain other types of multilayer network problems, as presented next.

## Illustrative examples: synthetic and real-world networks

To demonstrate application potential and illustrate implementation of the proposed framework, different examples of multilayer heterogeneous network flow are presented. The varied range of examples is thereby aimed at displaying generality and breadth of framework application. Further extensions related to domain specific applications are introduced as well.

**Figure 3:**
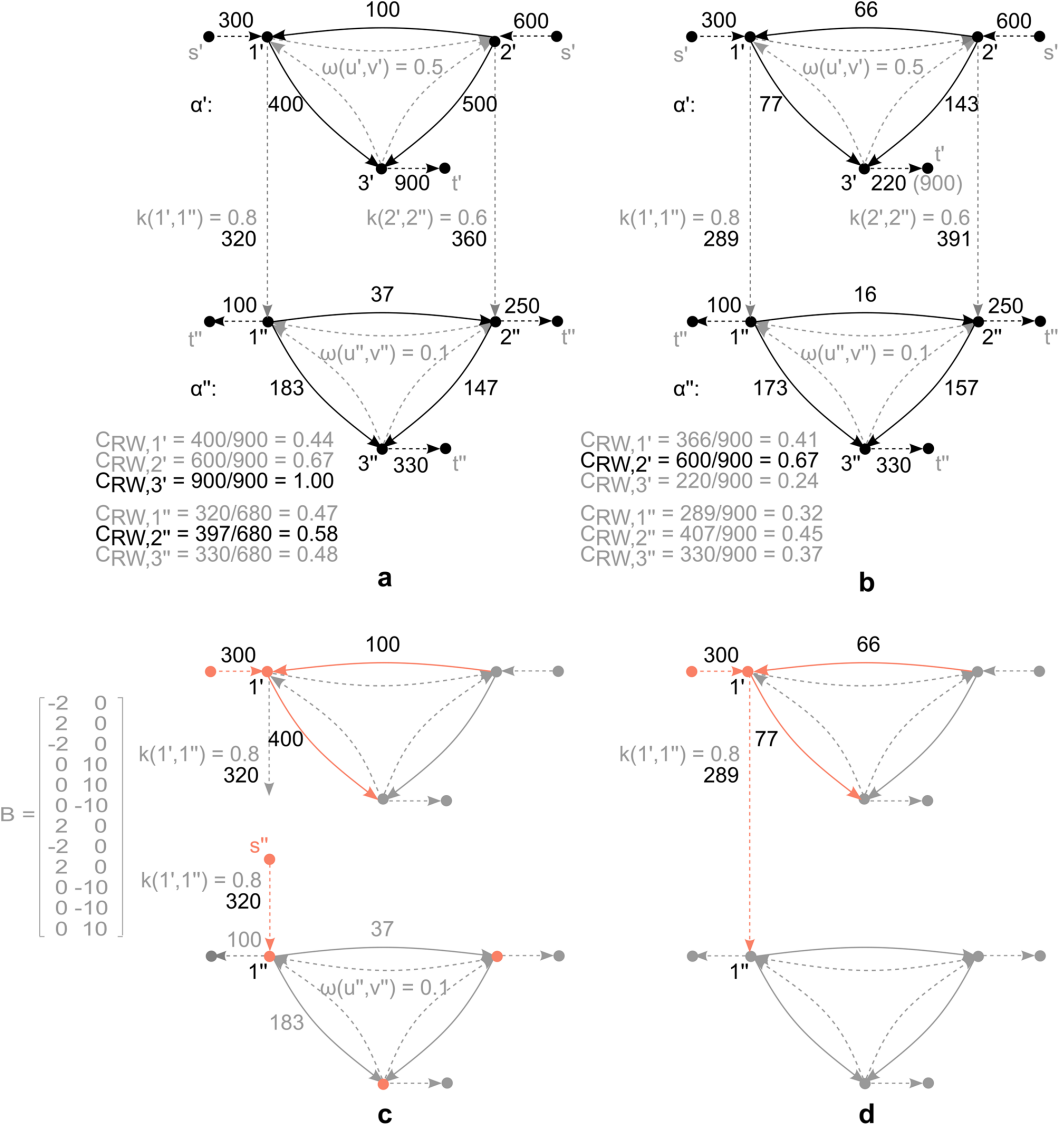
Simplified illustration of multilayer network flow derivation. (**a**) Illustrative multilayer flow network, comprising of two layers $\alpha '$, $\alpha ''$, single-source $s'$ and single-sinks $t'$, $t''$, where intra-layer flow is determined by arc weights $\omega '$, $\omega ''$, while inter-layer flow conversion is determined by conversion coefficient *k*. Multilayer network flow is derived as *heterogeneous network flow*, based on the proposed framework. The figure depicts, furthermore, the corresponding flow based random walk node betweenness centrality $C_{RW}$^44^, derived as node flow throughput, normalized by maximum *st*-flow (note that the measure is a function of network flow). Highest centrality denotes node(s) with highest impact, importance or influence. (**b**) Multilayer network flow derived as a simple mono-semantic random walk, based on^46^. The figure depicts, furthermore, derivation of corresponding flow based random walk centrality $C_{RW}$^44^, as proposed in^46^. By comparative analysis (panel a and b) a different network flow is observed. The difference is reflected in node centrality (highest impact/importance) as well, where removal of the highest centrality node in the first network (panel a: node $3'$ in layer $\alpha '$), would result in a collapse of the entire network, given removal of the only sink (demand) node in layer $\alpha '$ (reducing all flows to 0, due to flow balance and coupling constraints). In the second network (panel b), however, the removal of the highest centrality node would only result in a flow rebalance, not reflecting the true coupling nature of the underlying evolution process. (**c**) Flow conservation and coupling (as satisfied by the proposed framework in panel a), where inter-layer coupling does not participate in conservation of flow of the source layer. The coupling acts as exogenous flow with respect to the sink layer, influencing, along with network topology, the respective node potentials. The panel contains also an example of basis matrix *B* corresponding to the network in panel a. (**d**) Flow conservation (and coupling) inconsistent (violated), for simple mono-semantic random walk approach to multimodal flow derivation in panel b (note that arc $(1',1'')$ is an inter-layer arc, where incident end nodes correspond to different layer semantics).

### Laplacian flow

To demonstrate implementation of the proposed framework in uncovering importance of specific network components, an illustrative example of flow based *random walk node betweenness centrality* $C_{RW}$^44^ is presented. The Petri net flow relations (statement (i)–(iv), Remark 2) are here extended, to apply to a broader range of application domains, such as random walks, which correspond to a simple type of linear dynamic flow, also known as *Laplacian flow*^39,49^. The Petri net flow relations are thereby complemented by node potential balance (cycle space) conditions (along the lines of the notion of fundamental cycles over states^38^), in a form afforded by Definition 8, as

13 $$\begin{aligned} B^{T} \lambda = 0 \end{aligned}$$

where $B^{T} = {\tilde{B}}^{T} \text {diag}(w)$ is the weighted image of any basis ${\tilde{B}}$ of null-space $N(A_{t}^{i})$, for each (unweighted) incidence matrix $A_{t}^{i}$ of network *H* and relevant layer $i \in \{1,\ldots ,b\}$, while $w(u,v) = \omega (u,v)^{-1}$ is the *weighted length* of each intra-layer arc $(u,v) \in A_{{\bar{A}}} (t_{u,v} \in T)$. The condition is explicitly added using topological graph theory and the fundamental theorem of linear algebra^36^, i.e.: (i) the cycle space is a $\text {Ker}(A_{t}^{i})$, of dimension $m - n + 1$; (ii) the cut space is an $\text {Im}(A_{t}^{i^{T}})$, of dimension $n-1$; (iii) $\text {Ker}(A_{t}^{i}) \bot \text {Im}(A_{t}^{i^{T}})$ and $\text {Ker}(A_{t}^{i}) \oplus \text {Im}(A_{t}^{i^{T}}) = {\mathbb {R}}^{m}$. Relation (13) corresponds to a *cycle space condition* for all related cycles $h \in H_{A}$ of a weighted connected graph, where for an HFN of *b* layers, $|H_{A}| = |E_{A}| - |V_{M}| + 2 \cdot b$, as derived from^50^. The condition conforms to steady state requirements of a *donor-controlled compartmental system*^39^, where an arc flow is a function of mass *q* in the originating compartment *u*, for each $u \in V_{M} \setminus \{s,t\} (u \in P)$. The flow is governed by the rate of mass transfer, equivalent to $\omega$, and the amount of exogenous flow *U* from the environment i.e.,

14 $$\begin{aligned} {\dot{q}} = \Omega q + U \end{aligned}$$

where *q* is a donor mass vector and $\Omega$ a compartmental matrix which is Metzler i.e., $[\Omega ]_{u,v} = \omega (v,u)$, $u \ne v$, $[\Omega ]_{u,v} = -\sum _{r,u \ne r} \omega (u,r)$, $u = v$. It is worth pointing out that in a random walk arc weights are symmetric i.e., $\omega (u,v) = \omega (v,u)$ for each arc. The proposed framework, however, supports asymmetry in arc weights as well i.e., $\omega (u,v) \ne \omega (v,u)$, which is a more general case. Computational complexity of relation (13) is dictated by Gaussian elimination in producing the basis of the incidence matrix null-space, which has an arithmetic complexity of $O(n^{3})$, where *n* is the graph order, while time complexity is algorithm dependent and is polynomial (using standard algorithm forms). Overall, the proposed framework introduces a form of *multilayer Laplacian flow*. By comparison, state-of-the-art Laplacian flow applied to multilayer network structures^46^ does not capture the notion of coupling (conversion) between different semantic domains. It is hence not applicable to many physical problems which rely on laws of flow and mass conservation (e.g., energy networks, chemical processes, financial transactions, etc.), compared to the proposed framework, as demonstrated in the following example.

The multilayer network selected for the illustrative example (Fig. 3a), may in a broad sense represent a resource shipped from source $s'$ to sink $t'$ of a source layer $\alpha '$, according to an arc weight $\omega$ (interpretable also as transition probability, arc preference, or conductance). The shifting of the resource may generate further outputs in form of e.g., products, revenues, mechanical force or similar, with respect to the sink layer $\alpha ''$, obtained based on a conversion coefficient *k*. For the sake of generality, one or both layers may be assumed to meet (Eq. 13). The approach is thereby general enough to represent a number of equivalent problem formulations. In this particular case the object in Fig. 3a represents an energy network, comprising of a three tank energy resource layer $\alpha '$ (node $1',2'$ and $3'$), and a two generator power conversion layer $\alpha ''$ (node $1''$ and $2''$). Both layers have exogenous sources and/or sinks, referring to inflow (generation) or outflow (demand), respectively. The flow in both layers is a linear function of donor mass, where in layer $\alpha '$ the proportion refers to a constant discharge rate $\omega (u',v') = 0.5$, $\forall (u',v') \in A_{{\bar{A}}}, u',v' \in V_{M}'$, while in layer $\alpha ''$ it refers to conductance $\omega (u'',v'') = 0.1$, $\forall (u'',v'') \in A_{{\bar{A}}}, u'',v'' \in V_{M}''$ ($V_{M}' \cup V_{M}'' \subseteq V_{M}$, Definition 7). Coefficient *k* refers to a conversion constant.

#### Node centrality and flow interpretation

The state of each node in Fig. 3a can be determined from a steady state solution of the random walk evolution process, where based on^46^, *k* would be considered a weight $\omega$ (a form of layer switching probability), resulting in Fig. 3b. For the presented class of problems, however, the lack of approach^46^ lies in the fact that each edge of the multilayer network is given uniform treatment, and no distinction is made with respect to inter-layer coupling in the derivation of flow (compare Fig. 3c,d, relating to Fig. 3a,b, respectively). First of all, the induced inter-layer interaction is a flow conversion (Definition 9) and does not participate in conservation of flow of the source layer, but it does participate in overall conservation of mass. Secondly, the flow conversion acts as exogenous flow with respect to the sink layer, influencing, along with network topology, the respective state probabilities or node potentials of the sink (Fig. 3c). The outcome in the first place is a different result obtained for network flow (Fig. 3a), which directly affects derivation of the corresponding flow based centrality measure $C_{RW}$^44^, as

15 $$\begin{aligned} C_{RW}(u) = \sum \nolimits _{s,t \in V} \tau _{st}(u) \end{aligned}$$

where $\tau _{st}(u)$ corresponds to flow throughput of node *u*, normalized by maximum *st*-flow. The second difference relates to centrality measure interpretation (Fig. 3a,b), given that inter-layer mapping of flow has different effects on different layers, i.e.: it affects the corresponding sink layer (as exogenous flow), however, it does not affect the source layer it “originated” from. The difference is evident in obtained node centrality (impact/importance) rankings, where removal of the highest centrality node in Fig. 3a (node $3'$ in layer $\alpha '$) would result in a collapse of the entire network (given removal of the only sink or demand node in layer $\alpha '$, reducing all flows to 0, due to flow balance and coupling constraints). In Fig. 3b, however, the removal of the highest centrality node would result in a flow rebalance only, not reflecting the true coupling nature of the underlying evolution process. The proposed framework extends therefore differently the notion of flow based centrality to multilayer flow type networks, providing therewith a new perspective to multilayer flow based assessments.

To highlight the benefits of multilayer flow derivation and demonstrate properties of flow based centrality further, an additional comparative analysis is presented, with respect to a classic type of centrality i.e., *geodesic centrality*. As a convenient candidate to the flow based counterpart, classic *node betweenness centrality* is assessed. Node betweenness centrality^44^ is derived as $C_{BC}(u) = \sum \nolimits _{s \ne u \ne t \in V} \sigma _{st}(u)/\sigma _{st}$, or the share of times a node *u* participates in the number of shortest paths $\sigma _{st}$ between single-source *s* and single-sink *t* of a network, where a shortest path is obtained as a path of minimum weighted length, along a sequence of non-repeating nodes and edges. It is easy to see that each node in Fig. 3a,b participates equally in each shortest path of the network, resulting in a uniform node betweenness centrality $C_{BC}$ across nodes. Ultimately, no distinction is made between functional properties of nodes, where again the most critical node is not identified, as opposed to the ranking derived from flow based random walk node betweenness $C_{RW}$. It is here worth stressing that geodesic centrality, unlike flow based centrality, is concerned with topology only and follows a shortest path intuition. In most networks, however, transition does not unfold along geodesic trajectories and is often dictated by node potentials and exogenous forces, as demonstrated in Fig. 3c. To highlight the difference, the example is expanded with an assessment of the classic node betweenness centrality (as one type of geodesic centrality), to demonstrate disadvantages of disregarding flows in centrality evaluations of flow type problems.

**Figure 4:**
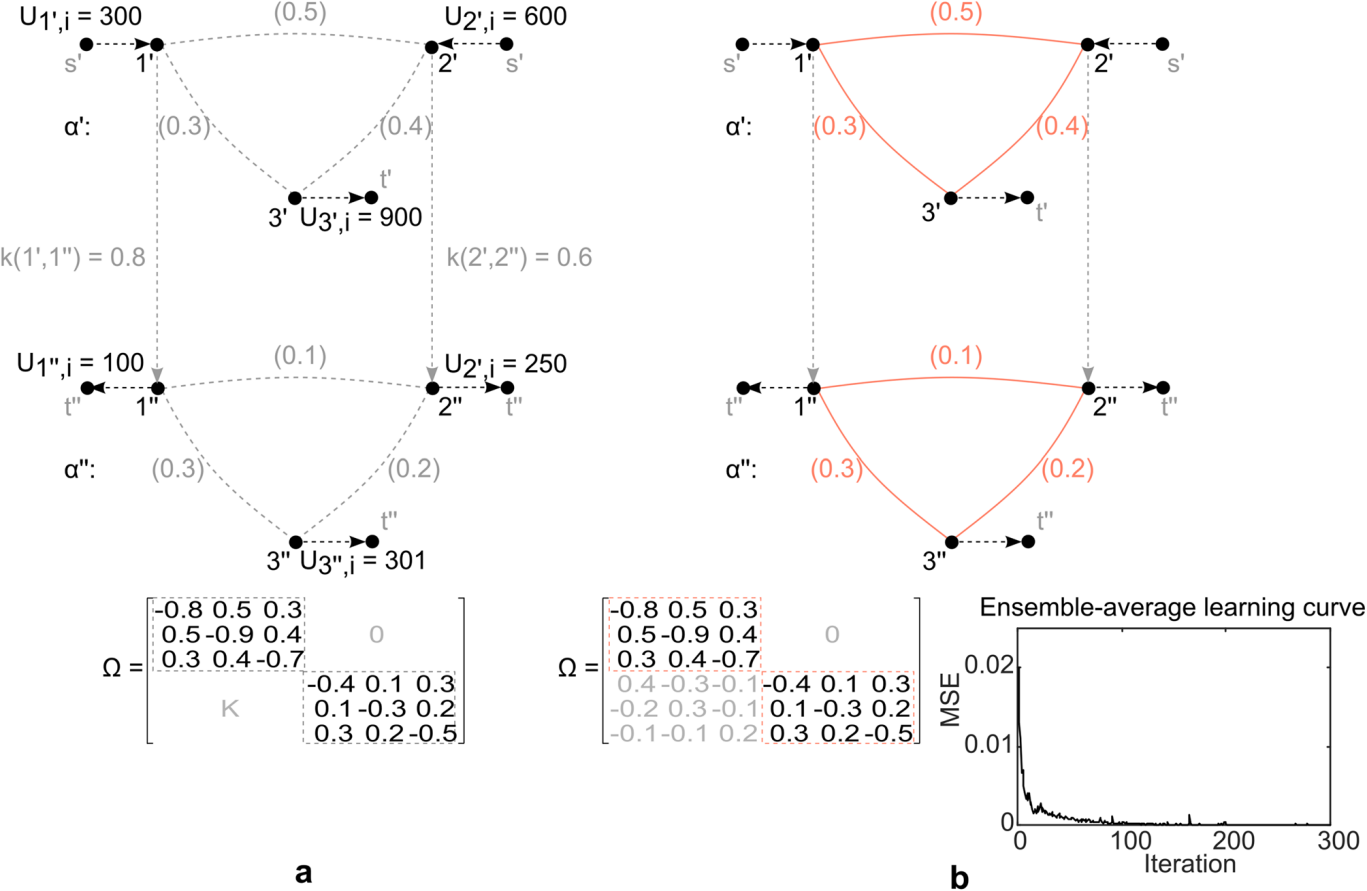
Simplified illustration of heterogeneous multilayer flow network (relationship) inference. (**a**) Illustrative heterogeneous multilayer flow network, with known features (nodes $\{1',\ldots ,3''\}$), and labelled outcomes (exogenous arcs $\{(s',1'),\ldots ,(3'',t'')\}$), by observation *i* (black), as well as unknown topology (grey) depicted as ground truth (corresponding to weights). Note that block matrix *K* is flow dependent (Eq. 18). (**b**) Derived multilayer network topology, based on LMS adaptive algorithm applied to dataset corresponding to panel a. Ensemble-average learning curve, $MSE_{i} = 1/R \sum _{R} [U_{i} + \Omega _{i-1} q_{i}]_{R}^{2}$^52^, corresponding to algorithm performance, obtained over 30 experiments *R*. Note that exogenous block entries of $\Omega$ (corresponding to *K*) relate to conversion of centred data. When solving for e.g., multilayer network flow, this would not pose a limitation, as one could derive the node potentials *q* based on the obtained weight matrix $\Omega$ (i.e., its pseudoinverse) and the given exogenous flow *U*. Ultimately, this would not distort the flow derivation, as potentials are fixed to within a constant (here average). Derivation of exact conversion coefficients *k*, however, would require additional constraints and a different approach, which is outside of scope for now.

### Graph learning—relationship inference

Application of the proposed framework can also be found in the domain of *graph learning*^51^, specifically in inference of multilayer relationships over multimodal data. Graph learning aims at uncovering structure in data through network inference, where *relationships* between data points are encoded in the network topology, while *data points* themselves are represented as node signals. Models can be statistical, physical or graph signal processing based^51^, where for the example described in this subsection a simple implementation of an adaptive gradient based steepest descent method is presented for illustrative purposes, to demonstrate application potential of the proposed framework. To this end, a synthetic dataset is derived based on Eq. (14) and Fig. 4a, where *q* corresponds to graph node signals perturbed by additive (uniformly distributed) zero-mean noise, while *U* refers to independent realizations of *labelled outcomes*, interpretable also as exogenous flow. The model of finding relationships $\Omega$ over features *q* can be formulated as a least mean square (LMS) or Widrow-Hoff adaptive algorithm^52^, where the optimal weigh matrix $\Omega$ solving $\min _{\omega } E|U +\Omega q|^{2}$ can be approximated via recursion

16 $$\begin{aligned} \Omega _{i} = \Omega _{i-1} - \mu [U_{i} + \Omega _{i-1} q_{i}] q_{i}^{T}, i \ge 0 \end{aligned}$$

such that $\Omega _{-1}$ is an initial guess, *i* an iteration step corresponding to observation *i*, and $\mu$ a sufficiently small constant step size. Feature vector $q_{i}$ and outcome vector $U_{i}$, are both centred and normalized by observation (ensemble) *i* for adaptive updates.

It is worth noting that, unlike a supra-Laplacian^2^, which is only an augmented Laplacian matrix over links and layers in flat form, the weight matrix $\Omega$ contains conversion ratios of the multimodal relationship as off-diagonal block elements, where

17 $$\begin{aligned} {[}\Omega ]_{u,v}= & {} {\left\{ \begin{array}{ll} -\sum \nolimits _{r,u \ne r} \omega (u,r), &{} \text {if } u = v\\ \omega (v,u), &{} \text {if } u \ne v \end{array}\right. } \end{aligned}$$

18 $$\begin{aligned} {[}\Omega ]_{{\tilde{u}},v}= & {} {\left\{ \begin{array}{ll} \sum \nolimits _{u} (k(u,{\tilde{u}}) \sum \nolimits _{r,u \ne r} \omega (u,r)), &{} \text {if } u = v, (u,{\tilde{u}}) \in A_{C}, q(u)> q(r)\\ \sum \nolimits _{u} k(u,{\tilde{u}}) \omega (v,u), &{} \text {if } u \ne v, (u,{\tilde{u}}) \in A_{C}, q(u) > q(v)\\ 0, &{} \text {otherwise} \end{array}\right. } \end{aligned}$$

such that inter-layer relationships participate in the derivation of the layer structure, as off-diagonal exogenous block entries (Eq. 18), while layers are identified from diagonal block matrices (Eq. 17) (Fig. 4b). The inference algorithm is consequently layer invariant, while identification of mono or multilayer structure resides in interpretation of the obtained weight matrix $\Omega$ (e.g., zero column and/or row block matrix sums, Fig. 4).

Ultimately, the proposed framework is capable of picking up a *layered* (exchange & conversion) as opposed to a *flat* (exchange only) relationship structure, in relationship inference performed on multimodal time series data. If, for instance, the six nodes in the example corresponded to financial time series, there would be no clear semantic feature distinction, yet a layered structure would exist (as layer 2, the bottom layer, accrues interest/exogenous inflow from layer 1, the top layer). It is worth noting that the relationships within each layer are different to the relationships between the layers, which can erroneously be omitted by a classic flat relationship inference approach. The proposed framework, however, enables capturing such complex network topology and the underlying relationships, without any a priori knowledge about the underlying data sets.

#### Algorithms and model interpretation

Overall, the LMS algorithm corresponds to a simple implementation of an adaptive gradient based steepest descent method, where signal statistics are replaced by suitable approximations (here instantaneous values) and convergence is dictated by step size. Different approximations may lead to different algorithms and performance^52^, where step size can be iteration dependent and regularization may be employed. The presented example is only illustrative, aimed at demonstrating capabilities of the proposed framework (where the performance of the implemented LMS algorithm could be further improved, though this is outside of the scope of the present manuscript). The key message conveyed is that the proposed framework opens up a new direction for graph learning based applications, by introducing a new type of mathematical object, where other types of relationship inference and graph learning algorithms will be explored separately, as part of future work.

It is here worth stressing that a form of graph learning over multilayer networks has been explored in recent work^53^, with reference to Structural Equation Models (SEMs), in form of multilayer SEMs (ml-SEMs). It is, however, worth noting that the proposed HFN framework is a generalization over^53^, where ml-SEMs represent a special case of HFNs, such that $\sum _{r^{i}, u^{i} \ne r^{i}} \omega (u^{i},r^{i}) = 1$. Where layers represent temporally lagged snapshots of a monoplex, the proposed framework can be interpreted as a Structural Vector Autoregressive Model (SVARM), as in^53^.

### Network robustness—U.S. GDP by industry

**Figure 5:**
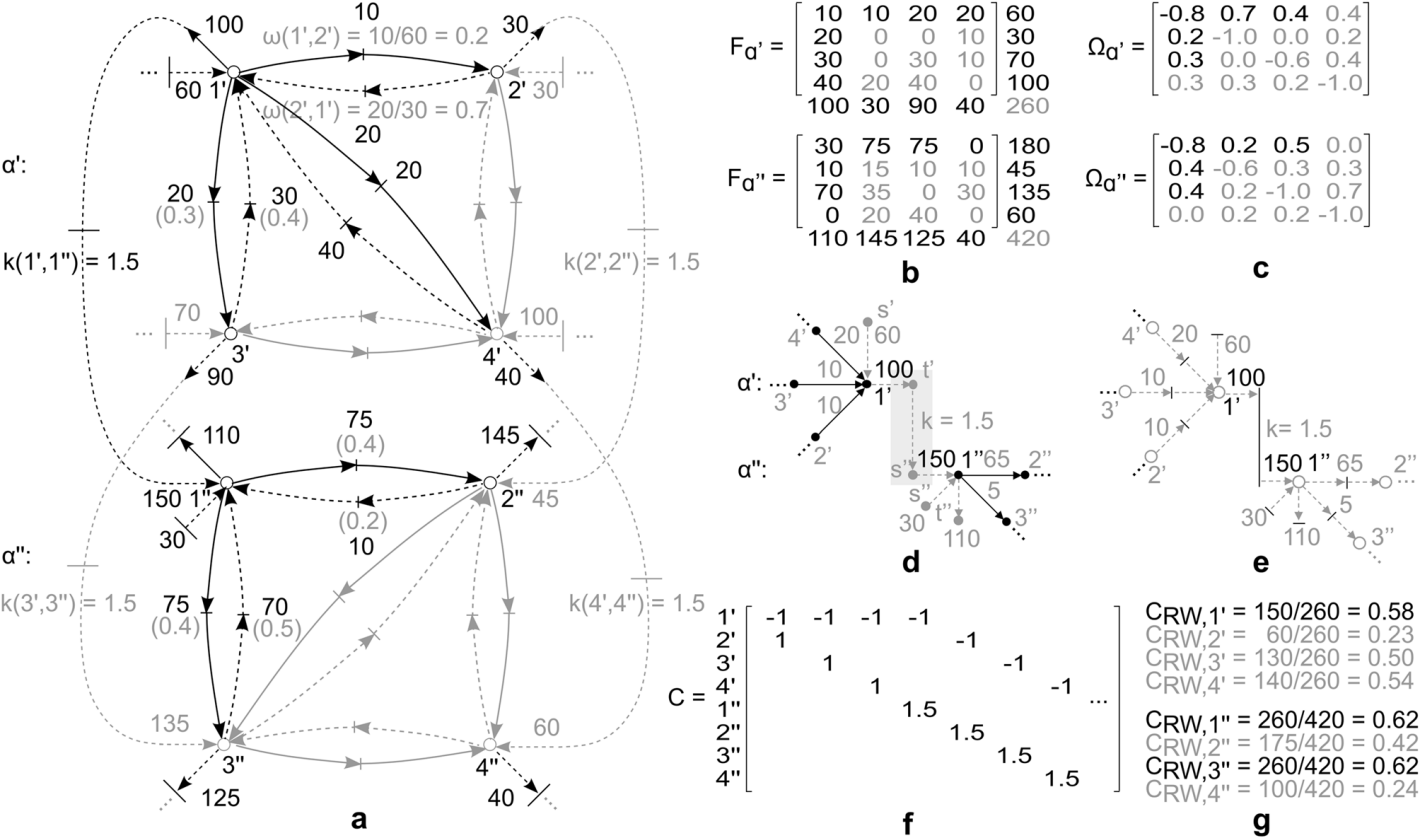
Simplified illustration of multilayer flow network (proxy), equivalent to the dataset of U.S. GDP by industry^54^. (**a**) Illustrative multilayer flow network interpretation, corresponding to dataset^54^. Nodes correspond to industries, while arcs and links correspond to economic activity (cross-industry and cross-layer network flow). Conversion coefficient *k* refers to a proportion defined with respect to e.g., rate of return, where layers $\alpha '$, $\alpha ''$ correspond to years (note that the network could be represented in compact form as well). Rate of transfer arc weights $\omega$ are derived from supply and cross-industry network flow. Note that a node (e.g., node $\{1'\}$), participates in domestic output and supply of commodities required by other industries (expressed in monetary terms), where total supply relates to node potential. The generated output participates, furthermore, in value added and capital carried over to the next time instance (i.e., node $\{1''\}$). (**b**) Illustration of cross-industry network flow matrix *F*, corresponding to dataset^54^ (as derived from the corresponding manual “Concepts and Methods of the U.S. Input-Output Accounts”, 2009^54^). The dataset consists of domestic supply (rows) and use (columns) of commodities by industry (intermediate use of commodities, net of contributions and subsidies). Note that^53^ relates total use, as opposed to total supply, to node potentials. (**c**) Compartmental weight matrix $\Omega$, derived from rate of transfer arc weights $\omega$. Note that $\sum _{r^{i}, u^{i} \ne r^{i}} \omega (u^{i},r^{i}) \ne 1$ (see weight matrix $\Omega$ diagonal) for some nodes (i.e., nodes $\{1',3',1'',2''\}$), as some funds get reinvested over time or further external investments are introduced (see flow matrix *F* diagonal). (**d**) HFN multilayer flow network excerpt (incident with nodes $\{1',1''\}$), as derived from panel b. (**e**) Petri net equivalent of HFN multilayer flow network excerpt incident with nodes $\{1',1''\}$. (**f**) Incidence (coupling) matrix *C*, encoding intra-layer and inter-layer relationships, corresponding to network in panel a. Note that weights (*Pre*, *Pos*) in the Petri net equivalent, are 1 for intra-layer (identity), and *k* for inter-layer conversion. (**g**) Example of flow based random walk node betweenness centrality $C_{RW}$ derivation, for nodes $\{1',\ldots ,4''\}$.

To demonstrate application of the proposed framework on a real-world multimodal flow network, the network of GDP by Industry, U.S. Bureau of Economic Analysis (BEA)^54^ is analysed, and also put into perspective with ml-SEM^53^. The dataset^54^ contains cross-industry use of commodities and GDP gross output by industry, over 71 industries, 21 industry groups, and a time range 1997–2019, expressed in millions of dollars. To simplify further exposition, a simple example (Fig. 5) is used as a guide throughout this subsection, in form of a proxy equivalent to^54^. The studied dataset (Fig. 5b) consists of domestic supply (rows) and use (columns) of commodities by industry (nodes), in form of a flow matrix *F*, by corresponding year $\alpha$. Supply relates to node potentials in this case, where rate of transfer (arc weight) $\omega$ is derived from cross-industry flow, as a share of compartmental (supply) outflow (Fig. 5a). Compartmental weight matrix $\Omega$ is derived from the rate of transfer $\omega$ (Fig. 5c), where $\sum _{r^{i}, u^{i} \ne r^{i}} \omega (u^{i},r^{i}) \ne 1$ for certain nodes (see $\Omega$ diagonal), as some funds get reinvested over time or further exogenous investments are introduced (see *F* diagonal). Inter-layer coupling corresponds to transformation of net output by industry, where conversion *k* refers to a proportion defined with respect to e.g., rate of return. Multilayer structure comes hence from economic activity (Fig. 5a,b,d), rather than industrial clustering suggested in^53^. Each layer can therewith be interpreted as a snapshot in time, or equivalently as a Petri net state (Fig.  5a,e,f).

To derive the exact conversion coefficient *k*, one requires information about the capital carried over from a previous time instance and the additional investment at a given time (compare nodes $1''$ and $2''$, Fig. 5a,b; both nodes have nonzero *F* diagonal entries). From there, *k* is derived as a proportion between exogenous outflow from the previous time instance and accrued capital in the next one (Fig. 5a,d). The value added may correspond to e.g., accrued interest, which would not be captured by a traditional approach, e.g.^46^. The proposed framework, in contrast, enables a multilayer flow interpretation of economic activity in^54^, uncovering some interesting properties.

#### Economic robustness and industry ranking

The derived network equivalent finds first of all interpretation in the domain of Stochastic Petri nets (SPN), where an SPN with given transition rates induces a Markov chain on its reachable set^38^. One can hence derive a corresponding steady state probability distribution, where elements refer to probability of being in a state $Q_{i}$ (here node potentials *q* on a layer $\alpha ^{i}$).

**Figure 6:**
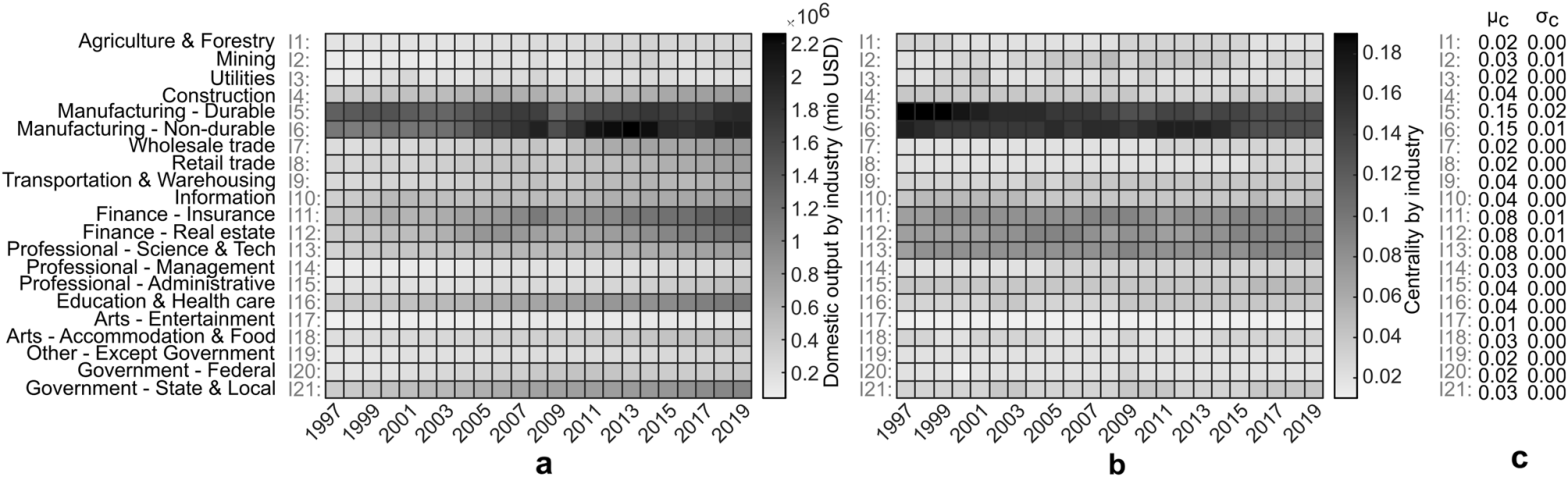
Illustration of random walk node betweenness centrality, for dataset^54^. (**a**) Heat map of domestic output by industry (net of contributions and subsidies), by year and 21 industry group, for dataset^54^. Note the fluctuations, particularly fall in domestic output for 2009 as a consequence of the 2008 economic crisis. (**b**) Heat map of random walk node betweenness centrality $C_{RW}$, by year and 21 industry group, for dataset^54^. Highest centrality is observed in 5 groups (Manufacturing—Durable, Non-durable; Finance—Insurance, Real estate; and Professional—Scientific & Technical services). The centrality highlights relative position of depicted industry groups, in terms of monetary throughput. (**c**) Mean, $\mu _{c}$, and standard deviation, $\sigma _{c}$, of centrality $C_{RW}$, over the time range 1997–2019, for all industry groups. The results demonstrate a consistent centrality over a 23 year time span (see $\sigma _{c}$), where relative importance of industries (i.e., their relative monetary throughput with respect to other industries, and even with respect to a different monetary volume and economic environment i.e., the 2008 economic crisis) remains absolutely the same, which suggests some form of relative network (i.e., economic activity) robustness.

One can, furthermore, derive a flow based random walk node betweenness centrality $C_{RW}$ for each industry, directly from cross-industry and cross-layer flow (Figs. 5g, 6b). It is here interesting to note that all industries exhibit a consistent centrality over a 23 year time span (Fig. 6b,c), regardless of domestic output fluctuation (Fig. 6a). This means that the relative importance of each industry i.e., the relative monetary throughput with respect to other industries and even with respect to a different monetary volume and economic environment (i.e., the 2008 economic crisis), remains absolutely the same, which suggests some form of relative network (i.e., economic activity) robustness. The industry ranking based on random walk node betweenness centrality $C_{RW}$ is also more stratified (Fig. 6b), as opposed to domestic output ranking alone (Fig. 6a), where it should be noted that the centrality ranking takes both, domestic output and supply of commodities by industry (Fig. 5a,g), into consideration. The proposed framework is compared with the real situation, and introduces a *multilayer network flow interpretation* of U.S. GDP by industry. The resulting node centralities, as a network theory tool, provide insights on the relative importance of each industrial sector, revealing consistency among relative positions of industries over a time period of more than two decades, and a finer more stratified ranking as compared to domestic output ranking alone. They identify therein five key industrial sectors (Fig. 6b), their relative positions in monetary throughput, as well as relative robustness under different economic environments (consistent centrality, Fig. 6b,c, regardless of domestic output fluctuation, Fig. 6a), where exogenous flows are interpretable as, and act in form of, *control inputs*. The proposed framework effectively *captures the role of nodes (industries)* as not necessarily generators of outputs (products) alone (as measured by the classic gross domestic output ranking, Fig. 6a), but also as suppliers of commodities required by other industries (as captured by the proposed framework and the implemented flow centrality ranking, Fig. 6b,c), providing entirely new insights on an economy, its industries, their importance, and relative robustness.

## Discussion and outlook

The presented examples demonstrate some of the benefits of multimodal flow derivation in complex network analysis. Inter-layer flow conversion takes place across network links, which act as exogenous control flows with respect to the sink layer. The multimodal flows are implicitly either coupled (across layers) or uncoupled (in intra-layer flow derivation, specifically with regard to cycle space conditions). This property lends some new perspective to network interdependency assessment. Furthermore, establishing correspondence between two apparently distinct conceptual domains brings new insights to both formalisms, as the tools and models of one can be used to develop an understand tools and models of the other, and vice versa. As previously mentioned, the heterogeneous flow network enables derivation of a layered relationship structure (corresponding to connectivity and conversion of node data), as opposed to a classic flat relationship structure (corresponding to connectivity of node data only). This enables a physical interpretation of models with complex interactions between different semantic domains (e.g., chemical processes, energy networks, logistics, finance, or any other form of conversion process relying on the laws of conservation), in the form of multilayer network flows. Disregarding such context may lead to erroneous interpretations, as demonstrated in the presented examples. The Petri net, on the other hand, enables flattening of the layered relationship structure (not to be confused with the latter, classic flat relationship representation). The construction simplifies computation, as it reduces the complex multilayer network to a form of bipartite graph (of resource and transition nodes), at the expense of losing semantics. As the proposed framework, however, establishes reversible correspondence between the two mathematical objects, interpretation is always preserved and outputs can uniquely be transformed from one mathematical object to the other. In a hypothetical ’ablation’ analysis sense, Petri nets are needed to facilitate computation, while heterogeneous multilayer flow networks provide interpretation and access to well-established network theory tools. Removing flow from the formulation would leave a very limited scope for quantitative assessments (i.e., disregarding node potentials and exogenous forces), which would inhibit reasoning, as it would remove the building blocks of the underlying relationship structure (e.g.: feature vectors and labelled data, respectively; node probabilities with respect to random walks on multilayer networks; mutual relationships between nodes and exogenous control flows; etc.). It is also worth adding that the proposed framework produces outputs that are analytically obtained and are fully interpretable.

In prospect, one of the anticipated goals is to give further attention to the extraction of the exact conversion coefficient *k* in graph learning based relationship inference (learning based function approximation) problems. From a synthesis point of view it is clear that, at least in some cases, *k* is uniquely determined. Further interesting insights could be obtained through possible integration of nonlinear relationships and dynamic topology considerations within the proposed framework, drawing on rich literature from current state-of-the-art.

## Conclusion

In this paper the formal notion of heterogeneous network flow is proposed, as a multilayer flow function aligned with the theory of network flow. A dynamic equivalence with the framework of Petri nets is established, as the baseline model of concurrent event systems. The Petri net flow relations are here extended, to possibly incorporate both fundamental equations of balance, namely: flow balance, which is integral to the Petri net model, and node potential balance (cycle space condition), which may arise in relation to specific application domains. Overall, the key property of the proposed framework lies in the ability to derive multimodal flows across different semantic domains, satisfying conditions of cross-layer conservation and coupling. The proposed framework provides therewith an extension to the recently introduced field of multilayer networks.

Some covered applications include multilayer Laplacian flow and multilayer flow centrality, as well as graph learning based inference of multilayer relationships over multimodal data. On synthetic data the proposed framework demonstrates benefits of multimodal flow derivation in critical component identification. It also displays applicability in relationship inference (learning based function approximation) performed on multimodal time series. On real-world data the proposed framework provides, among others, multimodal flow interpretation of U.S. economic activity, uncovering underlying empirical steady state probability distribution, as well as inherent network (economic) robustness.
